# *N*-(Quinolin-8-yl)quinoline-2-carbox­amide

**DOI:** 10.1107/S1600536812020144

**Published:** 2012-05-12

**Authors:** Yanfeng Li, Hongbo Zhou, Xiaoping Shen

**Affiliations:** a School of Chemistry and Chemical Engineering, Jiangsu University, Zhenjiang 212013, People’s Republic of China

## Abstract

In the title compound, C_19_H_13_N_3_O, the dihedral angle between the two quinoline systems is 11.54 (3)°. The mol­ecular conformation is stabilized by intra­molecular N—H⋯N and C—H⋯O hydrogen bonds, with N—H⋯N being bifurcated towards the two N atoms of the two quinoline rings. In the crystal, there are weak intermolecular π–π inter­actions present involving the quinoline rings [centroid–centroid distance 3.7351 (14) Å].

## Related literature

For the synthesis of the title compound and related structures, see: Kim *et al.* (2009 ▶). For applications of the title compound and background to the synthesis, see: Wang *et al.* (2011 ▶).
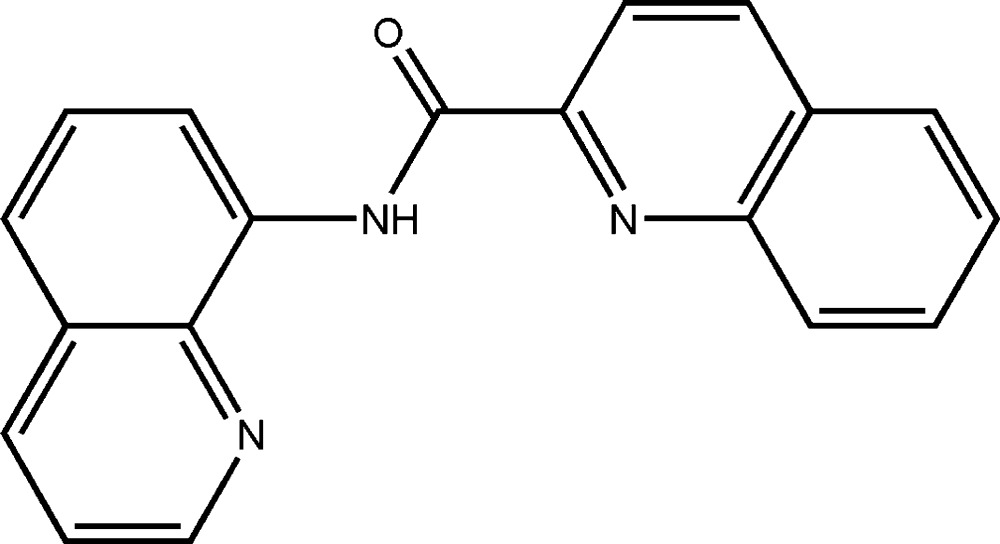


## Experimental

### 

#### Crystal data

C_19_H_13_N_3_O
*M* *_r_* = 299.32
Orthorhombic, 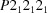

*a* = 6.3651 (13) Å
*b* = 11.475 (2) Å
*c* = 19.861 (4) Å
*V* = 1450.6 (5) Å^3^
*Z* = 4
Mo *K*α radiation
μ = 0.09 mm^−1^
*T* = 173 K
0.25 × 0.15 × 0.15 mm

#### Data collection

Rigaku Saturn 724 CCD diffractometer
Absorption correction: multi-scan (*ABSCOR*; Higashi, 1995 ▶) *T*_min_ = 0.978, *T*_max_ = 0.987
6769 measured reflections
1553 independent reflections
1442 reflections with *I* > 2σ(*I*)
*R*_int_ = 0.036

#### Refinement

*R*[*F* ^2^ > 2σ(*F* ^2^)] = 0.035
*wR*(*F* ^2^) = 0.082
*S* = 1.06
1553 reflections
208 parameters
H-atom parameters constrained
Δρ_max_ = 0.10 e Å^−3^
Δρ_min_ = −0.13 e Å^−3^

### 

Data collection: *CrystalClear* (Rigaku, 2008 ▶); cell refinement: *CrystalClear*; data reduction: *CrystalClear*; program(s) used to solve structure: *SHELXS97* (Sheldrick, 2008 ▶); program(s) used to refine structure: *SHELXL97* (Sheldrick, 2008 ▶); molecular graphics: *DIAMOND* (Brandenburg, 2006 ▶); software used to prepare material for publication: *SHELXTL* (Sheldrick, 2008 ▶).

## Supplementary Material

Crystal structure: contains datablock(s) I, global. DOI: 10.1107/S1600536812020144/zl2476sup1.cif

Structure factors: contains datablock(s) I. DOI: 10.1107/S1600536812020144/zl2476Isup2.hkl

Supplementary material file. DOI: 10.1107/S1600536812020144/zl2476Isup3.cml

Additional supplementary materials:  crystallographic information; 3D view; checkCIF report

## Figures and Tables

**Table 1:** Hydrogen-bond geometry (Å, °)

*D*—H⋯*A*	*D*—H	H⋯*A*	*D*⋯*A*	*D*—H⋯*A*
N2—H2*A*⋯N1	0.88	2.27	2.693 (2)	109
N2—H2*A*⋯N3	0.88	2.27	2.684 (2)	109
C12—H12⋯O1	0.95	2.25	2.867 (2)	122

**Fig. 1.**
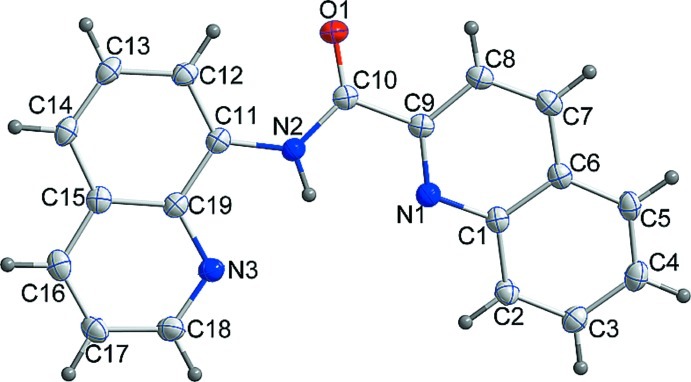
ORTEP diagram of the title compound with displacement ellipsoids drawn at the 30% probability level.

**Fig. 2.**
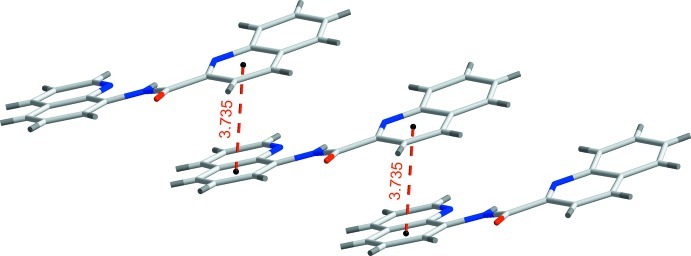
The crystal packing diagram of the title compound showing the intermolecular π-π interactions.The distances shown are between the centroids of the respective rings, and the symmetry operator codes for generating adjacent aromatic rings are -1+x, y, z and 1+x, y, z.

## supplementary crystallographic information

### Comment

The title compound (Hqcq) can act as a tridentate ligand, and has been incorporated into the cyanometalate building block [Fe(qcq)(CN)_3_]^-^ {qcq = 8-(2-quinolinecarboxamido)quinoline anion}, in which the Fe^III^ ion is coordinated by three carbon atoms of cyanide groups and three N-donors from the qcq ligand in a *mer*-arrangement (Kim *et al.*, 2009). Through replacement of the cyanide ligands the Fe(qcq) fragment can coordinate to transition metal ions to form various polynuclear and one-dimensional structures with fascinating magnetic properties such as single molecular magnets and single-chain magnets (Kim *et al.*, 2009; Wang *et al.*, 2011). Herein, the crystal structure of the tridentate ligand of Hqcq is presented.

The molecular structure of the title compound is shown in Fig. 1. The quinoline rings are essentially planar, with a maximum deviation of 0.046 (1) Å for atom C8 in the (N1/C1-C9) ring and 0.016 (1) Å for atom C14 in the (N3/C11-C19) ring. The dihedral angle between the two quinoline rings is 11.54 (3)°. The amide (N2/C10/O1) plane forms dihedral angles of 14.1 (1)° and 4.2 (1)° with the quinoline rings of (N1/C1-C9) and (N3/C11-C19), respectively. The bond lengths of the title molecule are slightly different from those reported for [Fe(qcq)(CN)_3_]^-^ (Kim *et al.*, 2009), probably owing to the coordination effect to the tridentate ligand. There are intramolecular hydrogen-bonding interactions between the amido N atom and the N atoms of the quinoline rings, and between the O atom of amide group and the C atom of the quinoline ring. The amido N atom (N2) forms bifurcated hydrogen bonds towards the two N atoms (N1, N3) of the two quinoline rings (Table 1).

In the crystal structure, no significant intermolecular hydrogen bonds are observed. The crystal structure features intermolecular π-π interactions between different types of quinoline rings with a distance of *ca*. 3.735 Å between the centroids of the respective rings (Fig. 2), and the adjacent rings tilted against each other.

### Experimental

The compound of 8-(2-quinolinecarboxamido)quinoline (Hqcq) was preparecd according to a literature method (Kim *et al.*, 2009). Then, 0.3 mmol of Hqcq was added to MeCN (20 mL) with stirring. The resulting solution was filtered and the filtrate was left for slow evaporation in the dark at room temperature. Yellow block-shaped crystals of the title compound suitable for single-crystal X-ray diffraction were obtained after two weeks. Melting point = 429.6-430.5 K. IR (KBr, cm^-1^): 3314(s), 3044(m), 1678(vs), 1523(vs), 1488(s), 1427(s), 1325(s), 1126(m), 913(s), 834(s), 764(vs), 611(m), 588(m).

### Refinement

All non-H atoms were refined with anisotropic thermal parameters. The C- and N-bound H atoms were calculated in idealized positions and included in the refinement in a riding mode (C-H = 0.95 Å, N-H = 0.88 Å) with *U*_iso_ for H assigned as 1.2 times *U*_eq_ of the attached atoms. In the absence of atoms heavier than Si and with Mo Kα radiation used Friedel-pair reflections have been merged (using a MERG 3 command) during the refinement. Assignment of the absolute structure is arbitrary.

### Crystal data

**Table:**

C_19_H_13_N_3_O	*F*(000) = 624
*M**_r_* = 299.32	*D*_x_ = 1.371 Mg m^−^^3^
Orthorhombic, *P*2_1_2_1_2_1_	Mo *K*α radiation, λ = 0.71073 Å
Hall symbol: P2ac2ab	Cell parameters from 6054 reflections
*a* = 6.3651 (13) Å	θ = 3.4–29.0°
*b* = 11.475 (2) Å	µ = 0.09 mm^−^^1^
*c* = 19.861 (4) Å	*T* = 173 K
*V* = 1450.6 (5) Å^3^	Block, yellow
*Z* = 4	0.25 × 0.15 × 0.15 mm

### Data collection

**Table:**

Rigaku Saturn 724 CCD diffractometer	1553 independent reflections
Radiation source: Rotating Anode	1442 reflections with *I* > 2σ(*I*)
Graphite monochromator	*R*_int_ = 0.036
φ and ω scans	θ_max_ = 25.3°, θ_min_ = 3.4°
Absorption correction: multi-scan (*ABSCOR*; Higashi, 1995)	*h* = −7→6
*T*_min_ = 0.978, *T*_max_ = 0.987	*k* = −13→13
6769 measured reflections	*l* = −23→18

### Refinement

**Table:**

Refinement on *F*^2^	Primary atom site location: structure-invariant direct methods
Least-squares matrix: full	Secondary atom site location: difference Fourier map
*R*[*F*^2^ > 2σ(*F*^2^)] = 0.035	Hydrogen site location: inferred from neighbouring sites
*wR*(*F*^2^) = 0.082	H-atom parameters constrained
*S* = 1.06	*w* = 1/[σ^2^(*F*_o_^2^) + (0.0494*P*)^2^] where *P* = (*F*_o_^2^ + 2*F*_c_^2^)/3
1553 reflections	(Δ/σ)_max_ < 0.001
208 parameters	Δρ_max_ = 0.10 e Å^−^^3^
0 restraints	Δρ_min_ = −0.13 e Å^−^^3^

### Special details

**Table:**

Geometry. All esds (except the esd in the dihedral angle between two l.s. planes) are estimated using the full covariance matrix. The cell esds are taken into account individually in the estimation of esds in distances, angles and torsion angles; correlations between esds in cell parameters are only used when they are defined by crystal symmetry. An approximate (isotropic) treatment of cell esds is used for estimating esds involving l.s. planes.
Refinement. Refinement of F^2^ against ALL reflections. The weighted R-factor wR and goodness of fit S are based on F^2^, conventional R-factors R are based on F, with F set to zero for negative F^2^. The threshold expression of F^2^ > 2sigma(F^2^) is used only for calculating R-factors(gt) etc. and is not relevant to the choice of reflections for refinement. R-factors based on F^2^ are statistically about twice as large as those based on F, and R- factors based on ALL data will be even larger.

### Fractional atomic coordinates and isotropic or equivalent isotropic displacement parameters (Å^2^)

**Table:**

	*x*	*y*	*z*	*U*_iso_*/*U*_eq_	
O1	−0.0149 (2)	0.41328 (13)	0.73413 (7)	0.0458 (4)	
N1	−0.1752 (2)	0.46907 (14)	0.56729 (8)	0.0328 (4)	
C1	−0.3327 (3)	0.44137 (16)	0.52317 (10)	0.0309 (4)	
N2	0.1160 (2)	0.54135 (14)	0.65635 (8)	0.0336 (4)	
H2A	0.0898	0.5707	0.6163	0.040*	
C2	−0.3099 (3)	0.47410 (18)	0.45477 (10)	0.0367 (5)	
H2	−0.1874	0.5146	0.4407	0.044*	
N3	0.3570 (3)	0.69181 (14)	0.58750 (8)	0.0385 (4)	
C3	−0.4630 (3)	0.44780 (19)	0.40896 (10)	0.0405 (5)	
H3	−0.4453	0.4691	0.3631	0.049*	
C4	−0.6456 (3)	0.38961 (18)	0.42926 (11)	0.0416 (5)	
H4	−0.7514	0.3724	0.3970	0.050*	
C5	−0.6737 (3)	0.35718 (18)	0.49498 (10)	0.0390 (5)	
H5	−0.7993	0.3187	0.5082	0.047*	
C6	−0.5164 (3)	0.38082 (16)	0.54318 (10)	0.0324 (5)	
C7	−0.5302 (3)	0.34377 (17)	0.61049 (10)	0.0364 (5)	
H7	−0.6505	0.3024	0.6257	0.044*	
C8	−0.3693 (3)	0.36763 (17)	0.65394 (10)	0.0358 (5)	
H8	−0.3739	0.3413	0.6993	0.043*	
C9	−0.1960 (3)	0.43222 (16)	0.62995 (10)	0.0317 (5)	
C10	−0.0232 (3)	0.46113 (17)	0.67895 (10)	0.0329 (4)	
C11	0.2965 (3)	0.58357 (17)	0.68860 (10)	0.0322 (5)	
C12	0.3571 (3)	0.55062 (19)	0.75238 (9)	0.0362 (5)	
H12	0.2716	0.4994	0.7781	0.043*	
C13	0.5465 (3)	0.59343 (19)	0.77909 (10)	0.0409 (6)	
H13	0.5880	0.5697	0.8229	0.049*	
C14	0.6721 (3)	0.66767 (18)	0.74419 (10)	0.0388 (5)	
H14	0.7999	0.6945	0.7635	0.047*	
C15	0.6127 (3)	0.70472 (17)	0.67950 (10)	0.0341 (5)	
C16	0.7326 (3)	0.78261 (18)	0.64020 (11)	0.0404 (5)	
H16	0.8604	0.8134	0.6572	0.049*	
C17	0.6643 (4)	0.81326 (18)	0.57795 (11)	0.0423 (5)	
H17	0.7423	0.8667	0.5513	0.051*	
C18	0.4767 (4)	0.76476 (18)	0.55348 (10)	0.0436 (6)	
H18	0.4330	0.7860	0.5094	0.052*	
C19	0.4227 (3)	0.66226 (17)	0.65098 (10)	0.0315 (4)	

### Atomic displacement parameters (Å^2^)

**Table:**

	*U*^11^	*U*^22^	*U*^33^	*U*^12^	*U*^13^	*U*^23^
O1	0.0481 (9)	0.0504 (9)	0.0389 (9)	−0.0030 (8)	−0.0031 (8)	0.0133 (7)
N1	0.0336 (9)	0.0318 (9)	0.0328 (9)	0.0016 (8)	0.0003 (8)	−0.0013 (7)
C1	0.0303 (10)	0.0286 (10)	0.0337 (10)	0.0012 (9)	0.0019 (9)	−0.0059 (8)
N2	0.0334 (9)	0.0372 (9)	0.0301 (8)	−0.0003 (8)	−0.0032 (8)	0.0028 (7)
C2	0.0339 (11)	0.0405 (12)	0.0356 (11)	−0.0021 (11)	0.0030 (9)	−0.0031 (9)
N3	0.0466 (10)	0.0354 (9)	0.0335 (9)	−0.0013 (9)	−0.0026 (9)	0.0011 (7)
C3	0.0426 (12)	0.0425 (12)	0.0366 (11)	0.0039 (11)	−0.0025 (10)	−0.0047 (10)
C4	0.0383 (12)	0.0374 (12)	0.0492 (14)	0.0031 (11)	−0.0089 (11)	−0.0080 (10)
C5	0.0342 (11)	0.0317 (11)	0.0512 (14)	−0.0025 (11)	−0.0032 (10)	−0.0044 (10)
C6	0.0317 (11)	0.0257 (10)	0.0397 (11)	0.0023 (9)	0.0021 (9)	−0.0040 (8)
C7	0.0357 (11)	0.0287 (10)	0.0448 (12)	0.0019 (10)	0.0075 (10)	−0.0004 (9)
C8	0.0393 (11)	0.0314 (10)	0.0367 (11)	0.0020 (10)	0.0046 (10)	0.0003 (9)
C9	0.0343 (11)	0.0276 (10)	0.0332 (11)	0.0046 (9)	0.0032 (9)	−0.0015 (8)
C10	0.0328 (11)	0.0327 (10)	0.0332 (10)	0.0056 (10)	0.0031 (9)	−0.0010 (9)
C11	0.0324 (11)	0.0337 (10)	0.0307 (11)	0.0043 (10)	−0.0002 (9)	−0.0053 (8)
C12	0.0385 (11)	0.0417 (12)	0.0283 (10)	0.0031 (11)	0.0018 (10)	−0.0015 (9)
C13	0.0418 (12)	0.0510 (14)	0.0300 (11)	0.0074 (12)	−0.0052 (10)	−0.0054 (9)
C14	0.0352 (11)	0.0462 (13)	0.0350 (12)	0.0071 (11)	−0.0055 (10)	−0.0104 (10)
C15	0.0360 (11)	0.0332 (10)	0.0330 (10)	0.0030 (10)	0.0024 (9)	−0.0092 (9)
C16	0.0402 (12)	0.0358 (11)	0.0454 (13)	−0.0030 (11)	0.0036 (10)	−0.0139 (10)
C17	0.0496 (14)	0.0350 (11)	0.0423 (13)	−0.0087 (11)	0.0051 (11)	−0.0044 (9)
C18	0.0563 (14)	0.0372 (12)	0.0374 (11)	−0.0050 (12)	−0.0009 (11)	0.0031 (9)
C19	0.0336 (10)	0.0313 (10)	0.0295 (10)	0.0053 (9)	0.0008 (9)	−0.0052 (8)

### Geometric parameters (Å, º)

**Table:**

O1—C10	1.227 (2)	C7—H7	0.9500
N1—C9	1.321 (2)	C8—C9	1.412 (3)
N1—C1	1.369 (2)	C8—H8	0.9500
C1—C2	1.417 (3)	C9—C10	1.505 (3)
C1—C6	1.417 (3)	C11—C12	1.377 (3)
N2—C10	1.354 (3)	C11—C19	1.421 (3)
N2—C11	1.402 (2)	C12—C13	1.406 (3)
N2—H2A	0.8800	C12—H12	0.9500
C2—C3	1.367 (3)	C13—C14	1.358 (3)
C2—H2	0.9500	C13—H13	0.9500
N3—C18	1.318 (3)	C14—C15	1.405 (3)
N3—C19	1.371 (2)	C14—H14	0.9500
C3—C4	1.400 (3)	C15—C16	1.411 (3)
C3—H3	0.9500	C15—C19	1.421 (3)
C4—C5	1.369 (3)	C16—C17	1.357 (3)
C4—H4	0.9500	C16—H16	0.9500
C5—C6	1.412 (3)	C17—C18	1.405 (3)
C5—H5	0.9500	C17—H17	0.9500
C6—C7	1.405 (3)	C18—H18	0.9500
C7—C8	1.367 (3)		
			
C9—N1—C1	117.09 (17)	C8—C9—C10	117.92 (17)
N1—C1—C2	118.50 (18)	O1—C10—N2	124.89 (19)
N1—C1—C6	122.59 (17)	O1—C10—C9	120.68 (19)
C2—C1—C6	118.90 (18)	N2—C10—C9	114.42 (16)
C10—N2—C11	128.30 (17)	C12—C11—N2	123.71 (19)
C10—N2—H2A	115.8	C12—C11—C19	120.01 (19)
C11—N2—H2A	115.8	N2—C11—C19	116.26 (17)
C3—C2—C1	120.5 (2)	C11—C12—C13	119.4 (2)
C3—C2—H2	119.8	C11—C12—H12	120.3
C1—C2—H2	119.8	C13—C12—H12	120.3
C18—N3—C19	116.89 (19)	C14—C13—C12	122.1 (2)
C2—C3—C4	120.37 (19)	C14—C13—H13	118.9
C2—C3—H3	119.8	C12—C13—H13	118.9
C4—C3—H3	119.8	C13—C14—C15	119.8 (2)
C5—C4—C3	120.8 (2)	C13—C14—H14	120.1
C5—C4—H4	119.6	C15—C14—H14	120.1
C3—C4—H4	119.6	C14—C15—C16	123.5 (2)
C4—C5—C6	120.1 (2)	C14—C15—C19	119.3 (2)
C4—C5—H5	119.9	C16—C15—C19	117.19 (18)
C6—C5—H5	119.9	C17—C16—C15	119.7 (2)
C7—C6—C5	122.86 (19)	C17—C16—H16	120.2
C7—C6—C1	117.81 (18)	C15—C16—H16	120.2
C5—C6—C1	119.30 (18)	C16—C17—C18	119.0 (2)
C8—C7—C6	119.55 (19)	C16—C17—H17	120.5
C8—C7—H7	120.2	C18—C17—H17	120.5
C6—C7—H7	120.2	N3—C18—C17	124.4 (2)
C7—C8—C9	118.52 (19)	N3—C18—H18	117.8
C7—C8—H8	120.7	C17—C18—H18	117.8
C9—C8—H8	120.7	N3—C19—C11	117.95 (18)
N1—C9—C8	124.35 (19)	N3—C19—C15	122.76 (19)
N1—C9—C10	117.73 (18)	C11—C19—C15	119.28 (18)

### Hydrogen-bond geometry (Å, º)

**Table:**

*D*—H···*A*	*D*—H	H···*A*	*D*···*A*	*D*—H···*A*
N2—H2*A*···N1	0.88	2.27	2.693 (2)	109
N2—H2*A*···N3	0.88	2.27	2.684 (2)	109
C12—H12···O1	0.95	2.25	2.867 (2)	122
